# 1-(2-Fluoro­benz­yl)quinolinium bis­(2-sulfanylidene-1,3-dithiole-4,5-dithiol­ato-κ^2^ *S*,*S*′)nickelate(III)

**DOI:** 10.1107/S1600536811014899

**Published:** 2011-04-29

**Authors:** Wen-Wen Shan, Peng Zhang, Xi-Ying Hu

**Affiliations:** a Institute of Environmental and Municipal Engineering, North China University of Water Conservancy and Electric Power, Zhengzhou 450011, People’s Republic of China

## Abstract

The crystal structure of the title compound, (C_16_H_13_FN)[Ni(C_3_S_5_)_2_], consists of Ni^III^ complex anions and 1-(2-fluoro­benz­yl)quinolinium (fbq) cations. In the complex anion, the Ni^III^ cation is chelated by two 2-sulfanylidene-1,3-dithiole-4,5-dithiol­ate (dmit) dianions in a distorted square-planar geometry; the two dmit mean planes are twisted with respect to each other at a dihedral angle of 8.44 (3)°. In the fbq cation, the dihedral angle between the benzene ring and the quinoline ring system is 80.57 (14)°. The centroid–centroid distance of 3.860 (5) Å between benzene rings indicates π–π stacking between adjacent fbq cations. The distance of 3.4958 (18) Å between the S atom and the centroid of the pyridine ring suggests the existence of a lone-pair–aromatic inter­action between the anion and the cation. A short S⋯S contact [3.387 (2) Å] is also observed in the crystal structure.

## Related literature

For the potential applications of bis­(dithiol­ate)–metal complexes, see: Cassoux (1999 ▶). For the lone-pair–aromatic inter­action, see: Egli & Sarkhel (2007 ▶). For the oxidation of Ni^II^ compounds, see: Cassoux *et al.* (1991 ▶). For the synthesis, see: Wang *et al.* (1998 ▶).
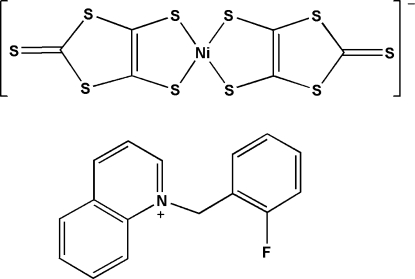


## Experimental

### 

#### Crystal data

(C_16_H_13_FN)[Ni(C_3_S_5_)_2_]
*M* *_r_* = 689.64
Triclinic, 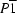

*a* = 8.740 (3) Å
*b* = 12.464 (4) Å
*c* = 12.464 (4) Å
α = 76.103 (4)°
β = 81.491 (6)°
γ = 81.491 (6)°
*V* = 1294.7 (8) Å^3^
*Z* = 2
Mo *K*α radiation
μ = 1.58 mm^−1^
*T* = 296 K
0.20 × 0.20 × 0.16 mm

#### Data collection

Bruker SMART APEXII CCD area-detector diffractometer
Absorption correction: multi-scan (*SADABS*; Bruker, 2005 ▶) *T*_min_ = 0.743, *T*_max_ = 0.786
6461 measured reflections
4475 independent reflections
2981 reflections with *I* > 2σ(*I*)
*R*_int_ = 0.054

#### Refinement

*R*[*F* ^2^ > 2σ(*F* ^2^)] = 0.052
*wR*(*F* ^2^) = 0.082
*S* = 0.99
4475 reflections
316 parameters
H-atom parameters constrained
Δρ_max_ = 0.47 e Å^−3^
Δρ_min_ = −0.38 e Å^−3^

### 

Data collection: *APEX2* (Bruker, 2005 ▶); cell refinement: *SAINT* (Bruker, 2005 ▶); data reduction: *SAINT*; program(s) used to solve structure: *SHELXTL* (Sheldrick, 2008 ▶); program(s) used to refine structure: *SHELXTL*; molecular graphics: *SHELXTL*; software used to prepare material for publication: *SHELXTL*.

## Supplementary Material

Crystal structure: contains datablocks I, global. DOI: 10.1107/S1600536811014899/xu5187sup1.cif

Structure factors: contains datablocks I. DOI: 10.1107/S1600536811014899/xu5187Isup2.hkl

Additional supplementary materials:  crystallographic information; 3D view; checkCIF report

## Figures and Tables

**Table 1:** Selected bond lengths (Å)

Ni1—S4	2.1529 (14)
Ni1—S5	2.1534 (15)
Ni1—S6	2.1502 (14)
Ni1—S7	2.1595 (14)

**Fig. 1.**
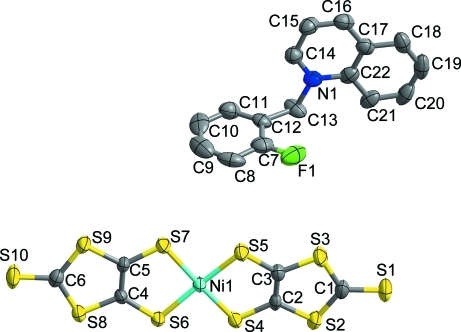
The cation and anion in [Fbzql][Ni(dmit)2], showing the atom-labelling scheme, with thermal ellipsoids drawn at the 50% probability level. Hydrogen atoms have been omitted for clarity.

**Fig. 2.**
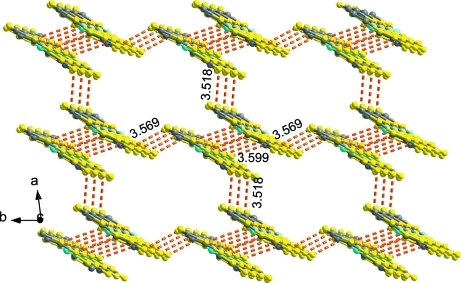
Three-dimensional supramolecular structure of [Ni(dmit)2]- anions through S···S contacts. Dashed lines indicate S···S interactions.

**Fig. 3.**
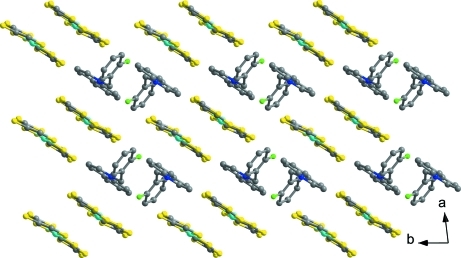
Packing of [Fbzql][Ni(dmit)2] viewed along c-axis

## supplementary crystallographic information

### Comment

The bis(dithiolate)-metal complexes and their analogues with interesting structures and/or potential applications such as conducting/magnetic or non-linear optical (NLO) materials have been reported in recent years (Cassoux, 1999). We report herein the crystal structure of the title bis-dithiolate-metal complex.

In this compound, the Ni^II^ cations of NiCl_2_.6H_2_O have been oxidized to Ni^III^ cation by I_2_ (Cassoux *et al.*, 1991), the Ni^III^ cation is coordinated with two dmit^II^ anions. As shown in Fig. 1, the asymmetric unit of the title compound contains one [Ni^III^(dmit)_2_]^-^ anion and one [Fbzql]^+^ cation. Each Ni^III^ ion is coordinated by four S atoms from two dmit ligands to complete a square-planar geometry, with Ni—S bond lengths ranging from 2.1502 (14) to 2.1595 (14) Å. Some of the [Ni^III^(dmit)_2_]^-^ anions are parallel and coplanar arrangements with the shortest S···S distance of 3.387 (2) Å (S3—S8^i^) [symmetry code: (i) *x*, *y*, -1 + *z*], indicating the existence of the S···S interactions. Adjacent [Ni^III^(dmit)_2_]^-^ anions are associated together through such S···S interactions result in a one-dimensional ribbon structure running along the *c*-axis. Two neighbouring anion ribbons are parallel each other and linked together through S6···S8^ii^ [symmetry code: (ii) 1 - *x*, -*y*, 2 - *z*] interactions forming a double-chain which is further connected to other four anion double-chains through S···S contacts [S2···S2^iii^: 3.519 (3) Å, S9···S9^iv^: 3.568 (2) Å [symmetry codes: (iii) -*x*, -*y*, 1 - *z*; (iv) 1 - *x*, 1 - *y*, 2 - *z*] along four orientations to form a three-dimensional supramolecular structure with large channels, as depicted in Fig 2.

Two [Fbzql]^+^ cations are associated together through face-to-face π···π interactions between two phenyl rings from different 2-fluorobenzyl groups (inter-centeriod distance: 3.8501 (9) Å) to form a bi-molecular unit, which expended to a one-dimensional structure running along the *c*-axis through another π···π interaction involving adjacent quinoline groups from different bi-molecular units with the shortest interface distance of 3.407 (4) Å.

The voids of the three-dimensional anion supramolecular structure are filled with the cation chains, as shown in Fig 3. Electrostatic attraction between the anions and cations play an important role in the stabilization of the whole structure. Additional investigation of this structure indicates that different noncovalent interactions can be detected between the two kinds of ions. The quinoline group of the [Fbzql]^+^ cation and neighbouring anion planes are parallel and associated together through lp···π (Egli & Sarkhel, 2007) interactions between one terminal sulfur atom of [Ni^III^(dmit)_2_]^-^ anion and the pyridine ring of the quinoline group [S9^v^-centroid distance 3.4958 (18) Å, symmetry codes: (v) 1 - *x*, 1 - *y*, 1 - *z*]. In addition, the distance between the benzene ring of the quinoline group from [Fbzql]^+^ cation and the terminal π system of adjacent [Ni^III^(dmit)_2_]^-^ anion is about 3.8294 (13) Å, indicating the existence of face-to-face π···π interaction which stabilizes the three-dimensional structure.

### Experimental

4,5-Bis(thiobenzoyl)-1,3-dithiole-2-thione (812 mg, 2.0 mmol; Wang *et al.*, 1998) was suspended in dry methanol (20 ml) and sodium (92 mg, 4.0 mmol) was added under a nitrogen atmosphere at room temperature to give a bright-red solution. NiCl_2_.6H_2_O (238 mg, 1 mmol) was then added, followed successively by I_2_ (127 mg, 0.5 mmol) and a solution of *N*-(2-fluorobenzyl)quinolinium chloride (274 mg, 1 mmol) in methanol at an interval of approximately 20 min. The solution was stirred for a further 30 min and the resulting solid collected by filtration. Single crystals of the title compound were obtained by evaporation of a dilute acetone solution over two weeks at room temperature.

### Refinement

H atoms were positioned geometrically and refined using a riding model, with C—H = 0.93-0.97 Å and *U*_iso_(H) = 1.2U_eq_(C).

### Crystal data

**Table:**

(C_16_H_13_FN)[Ni(C_3_S_5_)_2_]	*Z* = 2
*M**_r_* = 689.64	*F*(000) = 698
Triclinic, *P*1	*D*_x_ = 1.769 Mg m^−^^3^
Hall symbol: -P 1	Mo *K*α radiation, λ = 0.71073 Å
*a* = 8.740 (3) Å	Cell parameters from 717 reflections
*b* = 12.464 (4) Å	θ = 2.7–25.1°
*c* = 12.464 (4) Å	µ = 1.58 mm^−^^1^
α = 76.103 (4)°	*T* = 296 K
β = 81.491 (6)°	Needle, green
γ = 81.491 (6)°	0.20 × 0.20 × 0.16 mm
*V* = 1294.7 (8) Å^3^	

### Data collection

**Table:**

Bruker SMART APEXII CCD area-detector diffractometer	4475 independent reflections
Radiation source: fine-focus sealed tube	2981 reflections with *I* > 2σ(*I*)
graphite	*R*_int_ = 0.054
ω scans	θ_max_ = 25.0°, θ_min_ = 2.4°
Absorption correction: multi-scan (*SADABS*; Bruker, 2005)	*h* = −10→10
*T*_min_ = 0.743, *T*_max_ = 0.786	*k* = −12→14
6461 measured reflections	*l* = −11→14

### Refinement

**Table:**

Refinement on *F*^2^	Primary atom site location: structure-invariant direct methods
Least-squares matrix: full	Secondary atom site location: difference Fourier map
*R*[*F*^2^ > 2σ(*F*^2^)] = 0.052	Hydrogen site location: inferred from neighbouring sites
*wR*(*F*^2^) = 0.082	H-atom parameters constrained
*S* = 0.99	*w* = 1/[σ^2^(*F*_o_^2^) + (0.*P*)^2^] where *P* = (*F*_o_^2^ + 2*F*_c_^2^)/3
4475 reflections	(Δ/σ)_max_ < 0.001
316 parameters	Δρ_max_ = 0.47 e Å^−^^3^
0 restraints	Δρ_min_ = −0.38 e Å^−^^3^

### Special details

**Table:**

Geometry. All e.s.d.'s (except the e.s.d. in the dihedral angle between two l.s. planes) are estimated using the full covariance matrix. The cell e.s.d.'s are taken into account individually in the estimation of e.s.d.'s in distances, angles and torsion angles; correlations between e.s.d.'s in cell parameters are only used when they are defined by crystal symmetry. An approximate (isotropic) treatment of cell e.s.d.'s is used for estimating e.s.d.'s involving l.s. planes.
Refinement. Refinement of *F*^2^ against ALL reflections. The weighted *R*-factor *wR* and goodness of fit *S* are based on *F*^2^, conventional *R*-factors *R* are based on *F*, with *F* set to zero for negative *F*^2^. The threshold expression of *F*^2^ > σ(*F*^2^) is used only for calculating *R*-factors(gt) *etc*. and is not relevant to the choice of reflections for refinement. *R*-factors based on *F*^2^ are statistically about twice as large as those based on *F*, and *R*- factors based on ALL data will be even larger.

### Fractional atomic coordinates and isotropic or equivalent isotropic displacement parameters (Å^2^)

**Table:**

	*x*	*y*	*z*	*U*_iso_*/*U*_eq_	
Ni1	0.41132 (7)	0.16680 (5)	0.73700 (5)	0.03802 (18)	
C1	0.2375 (6)	0.0349 (4)	0.3914 (4)	0.0535 (14)	
C2	0.2836 (5)	0.0542 (3)	0.5856 (4)	0.0366 (12)	
C3	0.3561 (5)	0.1355 (4)	0.5140 (4)	0.0381 (12)	
C4	0.4629 (5)	0.1971 (3)	0.9616 (3)	0.0342 (11)	
C5	0.5169 (5)	0.2876 (3)	0.8906 (4)	0.0374 (12)	
C6	0.5638 (6)	0.3042 (4)	1.0876 (4)	0.0468 (13)	
C7	0.1790 (7)	0.5439 (5)	0.3914 (5)	0.0612 (16)	
C8	0.2756 (8)	0.5221 (5)	0.4752 (7)	0.084 (2)	
H8	0.3592	0.4662	0.4795	0.101*	
C9	0.2400 (10)	0.5875 (8)	0.5504 (7)	0.107 (3)	
H9	0.3006	0.5753	0.6085	0.129*	
C10	0.1210 (13)	0.6688 (7)	0.5439 (6)	0.112 (4)	
H10	0.1007	0.7125	0.5964	0.134*	
C11	0.0294 (9)	0.6875 (5)	0.4600 (6)	0.084 (2)	
H11	−0.0533	0.7440	0.4562	0.101*	
C12	0.0566 (6)	0.6248 (4)	0.3814 (4)	0.0469 (13)	
C13	−0.0480 (6)	0.6391 (5)	0.2939 (4)	0.0741 (19)	
H13A	−0.0599	0.5660	0.2839	0.089*	
H13B	−0.1499	0.6721	0.3202	0.089*	
C14	0.0848 (6)	0.7917 (4)	0.1797 (4)	0.0537 (14)	
H14	0.1111	0.8031	0.2454	0.064*	
C15	0.1308 (6)	0.8628 (4)	0.0803 (5)	0.0567 (15)	
H15	0.1856	0.9217	0.0791	0.068*	
C16	0.0947 (6)	0.8449 (4)	−0.0148 (4)	0.0556 (15)	
H16	0.1262	0.8916	−0.0822	0.067*	
C17	0.0105 (6)	0.7573 (4)	−0.0143 (4)	0.0487 (13)	
C18	−0.0288 (7)	0.7402 (5)	−0.1135 (5)	0.0735 (18)	
H18	0.0038	0.7850	−0.1818	0.088*	
C19	−0.1159 (8)	0.6565 (6)	−0.1074 (6)	0.087 (2)	
H19	−0.1426	0.6438	−0.1724	0.104*	
C20	−0.1649 (7)	0.5905 (5)	−0.0067 (7)	0.0769 (19)	
H20	−0.2242	0.5341	−0.0056	0.092*	
C21	−0.1301 (6)	0.6045 (4)	0.0913 (5)	0.0622 (16)	
H21	−0.1652	0.5590	0.1585	0.075*	
C22	−0.0391 (6)	0.6897 (4)	0.0882 (5)	0.0489 (14)	
F1	0.2101 (5)	0.4827 (3)	0.3141 (3)	0.1095 (14)	
N1	0.0047 (5)	0.7081 (3)	0.1846 (3)	0.0464 (11)	
S1	0.1815 (2)	−0.00195 (13)	0.28683 (12)	0.0827 (6)	
S2	0.19521 (17)	−0.03013 (11)	0.52769 (11)	0.0559 (4)	
S3	0.34297 (17)	0.14623 (11)	0.37414 (10)	0.0568 (4)	
S4	0.28115 (17)	0.03673 (10)	0.72632 (10)	0.0500 (4)	
S5	0.45265 (16)	0.22330 (10)	0.55899 (10)	0.0477 (4)	
S6	0.38493 (15)	0.10156 (9)	0.91397 (9)	0.0429 (3)	
S7	0.51306 (16)	0.31011 (10)	0.75026 (10)	0.0476 (4)	
S8	0.48219 (16)	0.18258 (10)	1.10106 (10)	0.0458 (3)	
S9	0.58815 (16)	0.37899 (10)	0.95174 (10)	0.0491 (4)	
S10	0.6115 (2)	0.34431 (13)	1.19276 (12)	0.0728 (5)	

### Atomic displacement parameters (Å^2^)

**Table:**

	*U*^11^	*U*^22^	*U*^33^	*U*^12^	*U*^13^	*U*^23^
Ni1	0.0479 (4)	0.0404 (3)	0.0299 (3)	−0.0144 (3)	−0.0087 (3)	−0.0077 (3)
C1	0.067 (4)	0.054 (3)	0.047 (3)	−0.004 (3)	−0.017 (3)	−0.023 (3)
C2	0.039 (3)	0.043 (3)	0.034 (3)	−0.007 (2)	−0.010 (2)	−0.014 (2)
C3	0.039 (3)	0.044 (3)	0.033 (3)	−0.008 (2)	−0.005 (2)	−0.011 (2)
C4	0.038 (3)	0.039 (3)	0.026 (3)	−0.005 (2)	−0.008 (2)	−0.005 (2)
C5	0.047 (3)	0.037 (3)	0.033 (3)	−0.010 (2)	−0.008 (2)	−0.011 (2)
C6	0.053 (3)	0.045 (3)	0.050 (3)	−0.005 (3)	−0.011 (3)	−0.021 (3)
C7	0.061 (4)	0.051 (4)	0.063 (4)	−0.015 (3)	0.004 (3)	0.001 (3)
C8	0.059 (5)	0.070 (5)	0.097 (6)	−0.011 (4)	−0.007 (4)	0.032 (4)
C9	0.123 (8)	0.128 (8)	0.072 (6)	−0.092 (7)	−0.027 (6)	0.029 (6)
C10	0.207 (12)	0.083 (6)	0.057 (5)	−0.079 (6)	0.012 (6)	−0.017 (5)
C11	0.114 (6)	0.064 (4)	0.064 (5)	−0.011 (4)	0.010 (4)	−0.007 (4)
C12	0.044 (3)	0.045 (3)	0.043 (3)	−0.013 (3)	0.000 (3)	0.008 (3)
C13	0.054 (4)	0.092 (4)	0.066 (4)	−0.035 (4)	−0.009 (3)	0.017 (3)
C14	0.059 (4)	0.052 (3)	0.051 (4)	−0.014 (3)	−0.003 (3)	−0.011 (3)
C15	0.064 (4)	0.045 (3)	0.060 (4)	−0.018 (3)	0.009 (3)	−0.013 (3)
C16	0.067 (4)	0.042 (3)	0.049 (4)	0.002 (3)	0.002 (3)	−0.002 (3)
C17	0.050 (3)	0.043 (3)	0.051 (4)	−0.001 (3)	−0.005 (3)	−0.008 (3)
C18	0.074 (5)	0.088 (5)	0.060 (4)	0.010 (4)	−0.019 (4)	−0.024 (4)
C19	0.079 (5)	0.106 (6)	0.092 (6)	0.024 (4)	−0.043 (5)	−0.056 (5)
C20	0.059 (4)	0.074 (4)	0.118 (6)	−0.005 (4)	−0.027 (4)	−0.052 (5)
C21	0.043 (4)	0.056 (4)	0.094 (5)	−0.008 (3)	−0.012 (3)	−0.024 (3)
C22	0.038 (3)	0.041 (3)	0.069 (4)	0.000 (3)	−0.011 (3)	−0.014 (3)
F1	0.135 (4)	0.070 (2)	0.118 (3)	−0.015 (2)	0.028 (3)	−0.034 (2)
N1	0.042 (3)	0.045 (2)	0.050 (3)	−0.015 (2)	−0.004 (2)	−0.001 (2)
S1	0.1301 (16)	0.0845 (11)	0.0519 (10)	−0.0306 (11)	−0.0308 (10)	−0.0277 (9)
S2	0.0773 (11)	0.0535 (8)	0.0478 (8)	−0.0257 (8)	−0.0199 (8)	−0.0137 (7)
S3	0.0775 (11)	0.0685 (9)	0.0306 (7)	−0.0251 (8)	−0.0035 (7)	−0.0149 (7)
S4	0.0716 (10)	0.0506 (8)	0.0327 (7)	−0.0289 (7)	−0.0140 (7)	−0.0010 (6)
S5	0.0626 (9)	0.0536 (8)	0.0320 (7)	−0.0263 (7)	−0.0031 (7)	−0.0088 (6)
S6	0.0614 (9)	0.0398 (7)	0.0322 (7)	−0.0198 (7)	−0.0112 (6)	−0.0050 (6)
S7	0.0675 (10)	0.0465 (7)	0.0334 (7)	−0.0260 (7)	−0.0087 (7)	−0.0047 (6)
S8	0.0609 (9)	0.0488 (8)	0.0315 (7)	−0.0120 (7)	−0.0095 (6)	−0.0106 (6)
S9	0.0645 (10)	0.0471 (8)	0.0446 (8)	−0.0213 (7)	−0.0110 (7)	−0.0161 (6)
S10	0.0957 (13)	0.0880 (11)	0.0528 (10)	−0.0297 (10)	−0.0184 (9)	−0.0335 (9)

### Geometric parameters (Å, °)

**Table:**

Ni1—S4	2.1529 (14)	C10—C11	1.366 (10)
Ni1—S5	2.1534 (15)	C10—H10	0.9300
Ni1—S6	2.1502 (14)	C11—C12	1.368 (7)
Ni1—S7	2.1595 (14)	C11—H11	0.9300
C1—S1	1.641 (5)	C12—C13	1.487 (7)
C1—S2	1.709 (5)	C13—N1	1.475 (6)
C1—S3	1.731 (5)	C13—H13A	0.9700
C2—C3	1.347 (6)	C13—H13B	0.9700
C2—S4	1.713 (4)	C14—N1	1.324 (5)
C2—S2	1.729 (4)	C14—C15	1.383 (6)
C3—S5	1.712 (4)	C14—H14	0.9300
C3—S3	1.735 (4)	C15—C16	1.346 (7)
C4—C5	1.354 (5)	C15—H15	0.9300
C4—S6	1.713 (4)	C16—C17	1.403 (6)
C4—S8	1.734 (4)	C16—H16	0.9300
C5—S7	1.708 (4)	C17—C22	1.399 (6)
C5—S9	1.742 (4)	C17—C18	1.403 (7)
C6—S10	1.637 (5)	C18—C19	1.362 (8)
C6—S9	1.725 (5)	C18—H18	0.9300
C6—S8	1.732 (4)	C19—C20	1.372 (8)
C7—F1	1.341 (6)	C19—H19	0.9300
C7—C12	1.355 (7)	C20—C21	1.357 (8)
C7—C8	1.390 (8)	C20—H20	0.9300
C8—C9	1.359 (9)	C21—C22	1.408 (6)
C8—H8	0.9300	C21—H21	0.9300
C9—C10	1.337 (10)	C22—N1	1.393 (6)
C9—H9	0.9300		
			
S6—Ni1—S4	86.54 (5)	N1—C13—H13A	108.4
S6—Ni1—S5	175.79 (6)	C12—C13—H13A	108.4
S4—Ni1—S5	93.15 (5)	N1—C13—H13B	108.4
S6—Ni1—S7	93.24 (5)	C12—C13—H13B	108.4
S4—Ni1—S7	172.57 (6)	H13A—C13—H13B	107.5
S5—Ni1—S7	87.61 (5)	N1—C14—C15	122.3 (5)
S1—C1—S2	123.9 (3)	N1—C14—H14	118.8
S1—C1—S3	122.9 (3)	C15—C14—H14	118.8
S2—C1—S3	113.2 (3)	C16—C15—C14	118.6 (5)
C3—C2—S4	120.8 (4)	C16—C15—H15	120.7
C3—C2—S2	116.6 (3)	C14—C15—H15	120.7
S4—C2—S2	122.6 (3)	C15—C16—C17	121.4 (5)
C2—C3—S5	121.8 (4)	C15—C16—H16	119.3
C2—C3—S3	115.6 (4)	C17—C16—H16	119.3
S5—C3—S3	122.5 (3)	C22—C17—C16	118.4 (5)
C5—C4—S6	121.0 (3)	C22—C17—C18	120.5 (5)
C5—C4—S8	116.3 (3)	C16—C17—C18	121.1 (5)
S6—C4—S8	122.7 (2)	C19—C18—C17	118.4 (6)
C4—C5—S7	121.6 (3)	C19—C18—H18	120.8
C4—C5—S9	115.7 (3)	C17—C18—H18	120.8
S7—C5—S9	122.7 (2)	C18—C19—C20	121.0 (6)
S10—C6—S9	123.7 (3)	C18—C19—H19	119.5
S10—C6—S8	123.5 (3)	C20—C19—H19	119.5
S9—C6—S8	112.8 (3)	C21—C20—C19	122.5 (6)
F1—C7—C12	117.6 (6)	C21—C20—H20	118.7
F1—C7—C8	118.2 (6)	C19—C20—H20	118.7
C12—C7—C8	124.2 (6)	C20—C21—C22	118.1 (6)
C9—C8—C7	115.7 (7)	C20—C21—H21	121.0
C9—C8—H8	122.1	C22—C21—H21	121.0
C7—C8—H8	122.1	C17—C22—N1	118.7 (5)
C10—C9—C8	122.4 (9)	C17—C22—C21	119.5 (5)
C10—C9—H9	118.8	N1—C22—C21	121.8 (5)
C8—C9—H9	118.8	C14—N1—C22	120.5 (4)
C9—C10—C11	119.9 (9)	C14—N1—C13	119.6 (4)
C9—C10—H10	120.1	C22—N1—C13	119.8 (4)
C11—C10—H10	120.1	C1—S2—C2	97.4 (2)
C12—C11—C10	121.4 (7)	C1—S3—C3	97.1 (2)
C12—C11—H11	119.3	C2—S4—Ni1	102.32 (15)
C10—C11—H11	119.3	C3—S5—Ni1	101.87 (15)
C7—C12—C11	116.4 (6)	C4—S6—Ni1	102.21 (14)
C7—C12—C13	121.4 (6)	C5—S7—Ni1	101.89 (14)
C11—C12—C13	122.1 (6)	C6—S8—C4	97.5 (2)
N1—C13—C12	115.4 (4)	C6—S9—C5	97.6 (2)
			
S4—C2—C3—S5	1.4 (6)	C17—C22—N1—C14	3.5 (7)
S2—C2—C3—S5	−178.4 (2)	C21—C22—N1—C14	−177.2 (4)
S4—C2—C3—S3	−177.8 (3)	C17—C22—N1—C13	179.1 (4)
S2—C2—C3—S3	2.4 (5)	C21—C22—N1—C13	−1.6 (7)
S6—C4—C5—S7	−1.0 (6)	C12—C13—N1—C14	−32.3 (7)
S8—C4—C5—S7	177.3 (3)	C12—C13—N1—C22	152.0 (5)
S6—C4—C5—S9	178.4 (2)	S1—C1—S2—C2	−178.3 (4)
S8—C4—C5—S9	−3.2 (5)	S3—C1—S2—C2	0.5 (3)
F1—C7—C8—C9	179.2 (5)	C3—C2—S2—C1	−1.7 (4)
C12—C7—C8—C9	0.4 (9)	S4—C2—S2—C1	178.4 (3)
C7—C8—C9—C10	−0.8 (10)	S1—C1—S3—C3	179.3 (4)
C8—C9—C10—C11	0.7 (12)	S2—C1—S3—C3	0.5 (3)
C9—C10—C11—C12	−0.2 (11)	C2—C3—S3—C1	−1.7 (4)
F1—C7—C12—C11	−178.7 (5)	S5—C3—S3—C1	179.1 (3)
C8—C7—C12—C11	0.0 (8)	C3—C2—S4—Ni1	−0.2 (4)
F1—C7—C12—C13	5.2 (7)	S2—C2—S4—Ni1	179.6 (2)
C8—C7—C12—C13	−176.1 (5)	S6—Ni1—S4—C2	−176.51 (17)
C10—C11—C12—C7	−0.1 (8)	S5—Ni1—S4—C2	−0.72 (17)
C10—C11—C12—C13	175.9 (5)	C2—C3—S5—Ni1	−1.8 (4)
C7—C12—C13—N1	−85.7 (6)	S3—C3—S5—Ni1	177.4 (3)
C11—C12—C13—N1	98.5 (6)	S4—Ni1—S5—C3	1.26 (17)
N1—C14—C15—C16	−1.1 (8)	S7—Ni1—S5—C3	−171.34 (17)
C14—C15—C16—C17	0.8 (8)	C5—C4—S6—Ni1	1.8 (4)
C15—C16—C17—C22	1.5 (8)	S8—C4—S6—Ni1	−176.5 (2)
C15—C16—C17—C18	179.0 (5)	S4—Ni1—S6—C4	−174.04 (16)
C22—C17—C18—C19	0.0 (8)	S7—Ni1—S6—C4	−1.47 (16)
C16—C17—C18—C19	−177.5 (5)	C4—C5—S7—Ni1	−0.3 (4)
C17—C18—C19—C20	0.3 (9)	S9—C5—S7—Ni1	−179.7 (3)
C18—C19—C20—C21	0.0 (10)	S6—Ni1—S7—C5	1.07 (17)
C19—C20—C21—C22	−0.5 (9)	S5—Ni1—S7—C5	−174.79 (17)
C16—C17—C22—N1	−3.6 (7)	S10—C6—S8—C4	179.3 (3)
C18—C17—C22—N1	178.9 (5)	S9—C6—S8—C4	−0.3 (3)
C16—C17—C22—C21	177.0 (5)	C5—C4—S8—C6	2.1 (4)
C18—C17—C22—C21	−0.5 (8)	S6—C4—S8—C6	−179.6 (3)
C20—C21—C22—C17	0.7 (8)	S10—C6—S9—C5	179.4 (3)
C20—C21—C22—N1	−178.6 (5)	S8—C6—S9—C5	−1.1 (3)
C15—C14—N1—C22	−1.1 (7)	C4—C5—S9—C6	2.6 (4)
C15—C14—N1—C13	−176.7 (5)	S7—C5—S9—C6	−177.9 (3)
